# Addition of monosodium glutamate can reduce the oxidative stability of lipids in pork burger patties via early-stage Maillard reaction products formation

**DOI:** 10.1016/j.crfs.2025.101091

**Published:** 2025-05-20

**Authors:** Arturo Auñon-Lopez, Verena Rohringer, Kübra Taranaci, Jon Alberdi-Cedeño, Marc Pignitter

**Affiliations:** a Institute of Physiological Chemistry, Faculty of Chemistry, University of Vienna, Josef-Holaubek-Platz 2, 1090, Vienna, Austria; b Vienna Doctoral School in Chemistry (DoSChem), Faculty of Chemistry, University of Vienna, Währinger Str. 42, 1090, Vienna, Austria; c Food Technology, Faculty of Pharmacy, Lascaray Research Center, University of the Basque Country (UPV-EHU), Paseo de la Universidad nº 7, 01006, Vitoria-Gasteiz, Spain

**Keywords:** Monosodium glutamate, Lipid oxidation, Protein oxidation, Pork patties, Maillard reaction, Schiff bases

## Abstract

Monosodium-L-glutamate (MSG) is an additive commonly used worldwide to increase the palatability of many foods, due to its popular umami flavor enhancing properties. While research has extensively focused on evaluating its safety and sensory properties, no prior studies have provided a comprehensive evaluation of the impact of MSG on the oxidative stability of food. Thus, this study investigated, for the first time, the influence of MSG supplementation (0.4 % and 1.2 %, w/w) on the progress of lipid and protein oxidation as well as the Maillard reaction in pork burger patties stored at 4 °C up to 4 days and cooked in an oven at 180 °C for 15 min. Analysis of lipidic extracts from the meat patties revealed the promotion of lipid oxidation during cooking due to 1.2 % MSG addition (e.g. > 53 % mean increase in alkanals), as determined by mass spectrometry and proton nuclear magnetic resonance. Conversely, 1.2 % MSG addition had no general impact on the formation of protein carbonyls and advanced glycation end products, although it promoted the generation of Schiff bases. This effect was attributed to the formation of N-dihydroxypropylideneglutamic acid, which was significantly correlated with the rise of secondary lipid oxidation products, suggesting that the prooxidant role of MSG might be related to the formation to this early-stage Maillard reaction product. Therefore, this study evidenced the interrelationship between oxidative processes and the Maillard reaction during MSG supplementation, highlighting the need for further research on the fate of this additive to ensure food quality and safety.

## Highlights

• Adding 1.2 % monosodium glutamate (MSG) to pork patties advanced lipid oxidation.
• The prooxidant effect of MSG was only visible when the patties were cooked.
• Generation of lipid-derived but not protein-derived carbonyls was promoted.
• MSG supplementation raised the content of early-stage Maillard reaction products.
• Formation of N-dihydroxypropylideneglutamic acid might be linked to these effects.

## Graphical abstract

**Figure:**
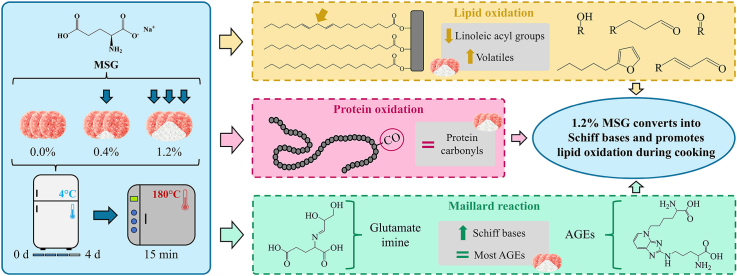

## 1 Introduction

The use of food additives is a common practice to increase the acceptability or the quality of many food products, whether by improving their flavor, adding color to them, or extending their shelf-life (Rangan and Barceloux, 2009; Jinap and Hajeb, 2010). Among these, glutamate and their salts are highly regarded, as they contribute to enhance the umami taste of different food products (Kos et al., 2023). In particular, monosodium-L-glutamate (MSG), E 621 in the European Union, is the most popular out of all glutamate salts, showing the highest umami flavor enhancing properties (Henry-Unaeze, 2017; Regulation, 2008). This additive, initially isolated by Kikunae Ikeda, provides a savory flavor to foods, resembling the taste of meat and broths (Ikeda, 2002). While glutamate in high quantities is naturally present in products such as cheese, tomatoes, and mushrooms, MSG is commonly used as a flavor enhancer to increase the palatability of a wide range of foods, including soups, stews, snacks, seafood, and meat (Jinap and Hajeb, 2010).

Since its introduction globally, the use of MSG over the years has been controversial, being associated with an unspecific set of symptoms known as the “MSG symptom complex” (Udom et al., 2024). This prompted an extensive line of research investigating the alleged health hazards of MSG supplementation. However, many of these studies were inconclusive, due to methodological and study design flaws, such as the use of excessive MSG doses that did not reflect its intake via food consumption (Zanfirescu et al., 2019). Thus, the direct cause-effect relationship between MSG intake and health issues could not be demonstrated reliably. As a result, MSG remains an authorized additive, and the U.S. Food and Drug Administration (FDA) currently considers it a “Generally Recognized As Safe” (GRAS) ingredient (World Health Organization, 2012).

Since then, several studies have evaluated the impact of the presence of MSG on the sensory characteristics and organoleptic properties of foods (dos Santos et al., 2014; Chun et al., 2014), with recent research works assessing the potential of MSG and other umami tasting compounds as sodium chloride replacers (De Leon et al., 2023; Maluly et al., 2017). However, studies investigating whether MSG can have an influence on the oxidative stability of food products are lacking. A few works have investigated the impact of MSG in oil mixtures or emulsions, showing different effects on their oxidative status depending on the conditions used or the presence of other additives (Bae and Kim, 2024; Wu et al., 2000). However, these results cannot be extrapolated to the wide range of foods in which MSG is used.

As a source of proteins and lipids, meat products are susceptible to both lipid and protein oxidation (Vicente and Pereira, 2024). One of the few studies that have explored the influence of MSG on the oxidative stability of meat found no impact on the development of rancidity in poultry meat when adding 0.3 % MSG (Jacobson and Koehler, 1970). However, thiobarbituric acid reactive substances were the only lipid oxidation parameter analyzed, and the MSG concentration used was notably below the currently established limit (Regulation, 2008). In addition, the Maillard reaction is another relevant process that takes place in meat and has an impact on its sensory properties and quality, sharing links with oxidative processes (Lin et al., 2023; Kou et al., 2024). While the umami enhancement properties of the Maillard reaction combined with MSG supplementation have been explored (Wang et al., 2024), little is known about the ability of MSG to directly participate in this reaction in meat.

Therefore, this study aimed to shed more light into the fate of MSG and its impact on the oxidative stability of meat at consumer relevant concentrations, using proton nuclear magnetic resonance (^1^H NMR), electron spin resonance (ESR), spectrophotometric assays, and mass spectrometry (MS) based techniques. Burger patties from pork was the food of choice, since this meat is often supplemented with MSG (Chun et al., 2014). Hence, pork patties were prepared, fortified with two different MSG concentrations (0.4 % and 1.2 %, w/w), and compared with the non-fortified controls after cooking, in order to evaluate the influence of MSG addition on the progress of lipid and protein oxidation, as well as Maillard reaction products (MRPs) formation. In addition, the patties were stored in a fridge prior to cooking for a different amount of days, to determine whether any MSG-associated effect was dependent on storage.

## 2 Materials and methods

### 2.1 Materials and chemicals

The ground pork used in this study was purchased from a local butcher shop (Vienna, Austria), whereas food grade MSG was obtained from Ajinomoto Foods Europe (Hamburg, Germany).

Anhydrous sodium sulfate, ethylene-glycol-bis(aminoethyl-ether)-N,N,N′,N'-tetraacetic acid (EGTA), guanidine hydrochloride, hydrochloric acid, L-arginine, L-lysine, monobasic sodium phosphate, MS grade ammonium formate, and perfluoropentanoic acid were acquired from Sigma-Aldrich (Vienna, Austria). Boric acid, chloroform, diethyl ether, ethyl acetate, isopropanol, magnesium chloride hexahydrate, petroleum ether, potassium chloride, sodium hydroxide, Tashiro's indicator, and trichloroacetic acid (TCA) were purchased from Carl Roth (Karlsruhe, Germany). Deuterium oxide as well as deuterated chloroform and methanol were obtained from Eurisotop (Tewksbury, MA, USA), while ethanol, sodium borate buffer, sulfuric acid, and the remaining MS grade chemicals (acetonitrile, formic acid, methanol, and water) were purchased from Avantor/VWR International (Vienna, Austria). Lastly, N(6)-carboxymethyllysine (CML), 2,4-dinitrophenylhydrazine (DNPH), and Kjeldahl tablets were acquired from Iris Biotech (Marktredwitz, Germany), AppliChem (Darmstadt, Germany), and Büchi AG (Flawil, Switzerland), respectively. Aside from the MS grade substances mentioned, all chemicals used were of analytical grade.

### 2.2 Preparation of meat burger patties

Pork burger patties were prepared as described by Botsoglou et al. (2014), using a Petri dish to shape portions of the purchased ground meat into 40 g patties, with average dimensions of 1 cm thickness and 5 cm diameter. A third of the patties were fortified with 0.4 % MSG (w/w), while 1.2 % MSG (w/w) was added to another third, before thoroughly mixing and molding them manually. These concentrations were chosen based on the common preferences of European consumers (Bellisle and France, 2008). The patties were then packed in plastic storage bags and kept in a fridge at 4 °C for 0, 2, 3, or 4 days, to then be subsequently cooked in an oven at 180 °C for 15 min. Afterwards, the patties were stored in a freezer at −80 °C until further use. In addition, one set of raw patties, with three replicates per MSG concentration group, was directly frozen at −80 °C without prior storage at 4 °C or cooking at 180 °C.

### 2.3 Extraction of hydrophilic and lipidic extracts from meat patties

Separation of the hydrophilic and lipidic components from the meat patties was done following the Bligh and Dyer extraction method (Bligh and Dyer, 1959), with modifications based on the protocols outlined by Pebriana et al. (2017) and Pérez-Palacios et al. (2008). After thawing the meat patties at room temperature, 5 g aliquots were taken and added to 15 mL methanol:chloroform 2:1 mix (v/v), followed by homogenization with a T18 brushless digital Ultra-Turrax (IKA®-Werke Labortechnik, Staufen, Germany) at 8000 rpm for 2 min. Each homogenate was then centrifuged (1250×*g*, 10 min) and filtered through 11 μm filter paper. A total of 15 mL chloroform was added to the remaining residue and the same homogenization, centrifugation, and filtration procedures were carried out. Both filtrates were then combined, mixed with 5 mL bidistilled water, shaken vigorously, and left standing until a clear biphasic system was formed.

On the one hand, the upper hydrophilic phase was transferred to another flask and concentrated via rotatory evaporation at room temperature, to remove the methanol, and freeze-drying in a VaCo 5-II freeze dryer (Zirbus Technology, Bad Grund, Germany), to remove the remaining water. The residue was then reconstituted in 1 mL methanol. On the other hand, the lower organic phase was dried with 1.5 ± 0.5 g anhydrous sodium sulfate and centrifuged (1250×*g*, 1 min), and the supernatant was concentrated with a rotatory evaporator at room temperature, obtaining the lipidic meat extracts. Both hydrophilic and lipidic extracts were stored at −20 °C until analysis.

### 2.4 Quantification of MSG in the hydrophilic extracts by targeted tandem mass spectrometry (MS/MS)

To determine the amount of MSG present in the hydrophilic extracts of the meat patties, these extracts were diluted with MS grade water in a 1:2 v/v ratio for 0.0 % added MSG groups and in a 1:10 v/v ratio for 0.4 % and 1.2 % MSG groups. Samples were then filtered with 0.20 μm polyvinylidene fluoride (PVDF) syringe filters and 10 μL were directly injected into a triple quadrupole LCMS-8040 mass spectrometer (Shimadzu, Korneuburg, Austria), with an electrospray (ESI) source operating in positive mode, with the following settings: nebulizing gas flow 3 L/min (N_2_), drying gas flow 10 L/min (N_2_), heat block temperature 350 °C, and desolvation line (DL) temperature 150 °C. Multiple reaction monitoring (MRM) mode was used for the MS/MS analysis, using argon as collision-induced dissociation (CID) gas, set at 230 kPa. The MRM transitions used for the determination of MSG (Table S1) were confirmed with the measurement of an external standard, and quantification was carried out using an external calibration curve (Table S2). Data analysis was carried out using Shimadzu LabSolutions 5.99 SP2.

### 2.5 Analysis of meat extracts by proton nuclear magnetic resonance (^1^H NMR)

The hydrophilic and lipidic extracts obtained in section 2.3 were analyzed with a Bruker Avance 400 spectrometer (Billerica, US) operating at 400 MHz. Prior to analysis, 150 μL hydrophilic extract were dried and reconstituted with 600 μL deuterated methanol, whereas 200 μL lipidic extract were added to 400 μL deuterated chloroform. Both deuterated solvents had a small amount of tetramethylsilane (0.03 %), which was used as internal reference. The ^1^H NMR spectrum of each sample was acquired using the same parameters as in a previous study (Martínez-Yusta and Guillén, 2014): spectral width 6410 Hz, pulse width 90°, acquisition time 4.8190 s, relaxation delay 3 s, number of scans 64, and total acquisition time 8 min 38 s.

The spectra were analyzed using MestreNova (Mestrelab Research, Santiago de Compostela, Spain), after Fourier transform, baseline correction, and phase correction, taking into consideration that the areas of the signals were proportional to the number of protons that generated them. The identification and quantification of linoleic acyl groups and lipid oxidation products, as well as the identification of the glutamate signals, were carried out based on previous research (Martínez-Yusta and Guillén, 2014; Guillén and Uriarte, 2012; Ijare et al., 2019; Wishart et al., 2009), with the latter being additionally confirmed with a MSG standard.

### 2.6 Analysis of volatile lipid oxidation products by solid phase microextraction followed by gas chromatography-mass spectrometry (SPME/GC-MS)

The analysis of secondary volatile lipid oxidation compounds was carried out directly from the meat samples, with a procedure described in a previous study (Alberdi-Cedeño et al., 2020). Thus, 1.5 g patty were introduced in a 20 mL headspace brown glass vial and SPME was performed, using a fiber coated with divinylbenzene/carboxen/polydimethylsiloxane (50/30 μm film thickness, 1 cm length) to extract the volatile compounds for 55 min at 50 °C, after a 5 min equilibration period. The extracted compounds were subsequently injected into a Shimadzu GC-QP2010 Ultra in splitless mode, with a 10 min desorption time and a 5 min purge time, with the injector set at 205 °C. Separation was achieved using a Phenomenex Zebron ZB-5 ms column (30 m × 0.25 mm × 0.25 μm, Aschaffenburg, Germany) as stationary phase and helium as carrier gas at a constant pressure of 117 kPa. A temperature gradient was applied, where the oven was initially kept at 50 °C for 5 min, followed by a temperature increase to 300 °C at a 4 °C/min rate and a hold period at 300 °C for another 30 min. The interface temperature was 305 °C, and the ion source and quadrupole mass analyzer were set to 230 °C and 150 °C, respectively. An ionization energy of 70 eV was used, and the mass range analyzed was *m/z* 40–600. Identification was done by mass spectra matching with a commercial database (NIST ver. 11.0), accepting an identity score equal or higher than 85 %. As the aim of this study was the comparison between the samples, semi-quantification was conducted based on the area counts of the base peak in the mass spectrum of each compound. When the base peak of two compounds overlapped, an alternative ion peak was selected for them.

### 2.7 Determination of protein carbonyls content by DNPH assay

The content of protein carbonyls in the pork patties, as a marker of protein oxidation, was evaluated following the guidelines from previous studies (Armenteros et al., 2009; Levine et al., 1994). A total of 5 mL pyrophosphate buffer (pH = 7.4) containing 100 mM potassium chloride, 10 mM tris-maleate, 2 mM sodium pyrophosphate, 2 mM magnesium chloride, and 2 mM EGTA, was added to 0.5 g patties, which were then homogenized with a T18 brushless digital Ultra-Turrax for 30 s at 16800 rpm. A 50 μL aliquot from each homogenate was then subjected to protein precipitation, adding 0.5 mL 10 % TCA to it. After centrifugation (5000×*g*, 5 min), the supernatant was removed and the pellet was incubated for 1 h at room temperature with 0.5 mL 0.2 % DNPH (w/v) solution in 2 N hydrochloric acid, shaking it every 15 min. Afterwards, a second protein precipitation with 0.5 mL 10 % TCA was performed, and the pellet was subsequently washed three times with 1 mL ethanol:ethyl acetate 1:1 mix (v/v), followed by vigorous mixing and another centrifugation (10000×*g*, 5 min). The supernatant was then removed and the pellet was left to dry for 30 min, after which it was dissolved in 750 μL 20 mM sodium phosphate buffer (pH = 6.5) with 6 M guanidine hydrochloride. Lastly, after mixing, the solution was centrifuged once more (5000×*g*, 2 min) and the supernatant was transferred to a quartz glass cuvette, in order to measure their absorbance at 280 nm and 370 nm with a Spark® Tecan spectrophotometric device (Männedorf, Switzerland) at 25 °C. The content of protein carbonyls was calculated from the absorbances as described by Levine et al. (1994), expressing the results in nmol carbonyls/mg protein.

### 2.8 Determination of stable radicals in meat by electron spin resonance (ESR)

Aiming to gather further insights into the progression of oxidative processes among the meat samples, the content of stable radicals in the meat was analyzed according to Yu et al. (2016), with slight modifications. Briefly, aliquots from the meat patties were freeze-dried in a Zirbus VaCo 5-II freeze dryer and placed in 4 mm ESR quartz tubes. ESR measurements were carried out in a Bruker X-band Elexsys-II E500 CW-EPR spectrometer, using the following parameters: center field 3505 G, modulation frequency 100 kHz, modulation amplitude 10 G, receiver gain 60 dB, attenuation 10 dB, sweep time 60 s, sweep width 200 G, averaged scan 3, conversion time 59 ms, microwave power 20 mW, and microwave frequency 9.43 GHz. The acquired spectra were analyzed with the Bruker XEPR software, allowing to integrate the singlet signal detected (g-factor = 2.006) and calculate the spin count in the meat samples, as described previously (Höfer and Carl, 2009; Zaunschirm et al., 2018).

### 2.9 Analysis of protein-bound advanced glycation end products (AGEs) by liquid chromatography-tandem mass spectrometry (LC-MS/MS)

The content of selected lysine- and arginine-derived AGEs was determined in the cooked meat samples, following the acid hydrolysis procedure outlined by Teerlink et al. (2004), with the modifications described by Auñon-Lopez et al. (2025). After preparation and filtration through 0.20 μm nylon syringe filters into high performance liquid chromatography (HPLC) vials, 10 μL sample were injected into a Shimadzu LCMS-8040 system and separated on a Luna HILIC 200 Å column (100 mm × 3 mm × 3 μm, Phenomenex), equipped with a HILIC precolumn (4 mm × 2 mm × 3 μm, Phenomenex). A buffer containing 6 mmol/L ammonium formate and 0.1 % formic acid (v/v) in water (pH = 2.4) was mobile phase A, while mobile phase B consisted of 80 % acetonitrile with 0.1 % formic acid (v/v) and 20 % mobile phase A (v/v). A constant flow rate of 0.4 mL/min was set, and the mobile phase gradient was as follows: 90 % B set for the first 2 min, 87 % B between minute 3 to minute 5, 30 % B between minute 9 to minute 15, and 90 % B between minute 20 to minute 30. The oven temperature was 25 °C.

MS settings were the same as the ones described in section 2.4, with the exception of a drying gas flow of 13 L/min (N_2_) and a DL temperature of 170 °C. MRM analyses were carried out in positive mode, with the MS transitions listed in Table S1, obtained from previous studies (Lin et al., 2023; Zhang et al., 2011; Baye et al., 2019; Gómez-Ariza et al., 2005; Liu et al., 2019; Martens-Lobenhoffer and Bode-Böger, 2012). For data analysis, MacCoss Lab Software Skyline 23.0 was used (MacLean et al., 2010). The content of CML and the amino acids lysine and arginine was quantified using external standard curves and expressed in relation to the protein content in the meat, which was determined via the Kjeldahl Method, as described by Mihaljev et al. (2015). The rest of the AGEs analyzed were compared in terms of area under curve.

### 2.10 Determination of schiff bases content

The content of Schiff bases in the meat was determined as described by (Utrera et al., 2012). In short, 1 g patties were homogenized in 10 mL 20 mM sodium phosphate buffer (pH = 6.5) using a T18 brushless digital Ultra-Turrax (8000 rpm, 30 s) and filtered. Subsequently, 200 μL of a 1:20 dilution of each filtrate (v/v) were transferred to a 96-well plate and their fluorescence emission spectrum between 370 nm and 500 nm was acquired, using a FlexStation microplate reader (Molecular Devices, San Jose, CA, USA), with a wavelength increment of 5 nm and an excitation wavelength of 350 nm. The Schiff bases content was expressed as fluorescence intensity units at 460 nm.

### 2.11 Untargeted analysis of polar lipid extracts by HPLC coupled to high resolution-mass spectrometry (HRMS/MS)

To detect the presence of oxidized triacylglycerols as well as other possible molecules of interest within the meat lipids, a solid phase extraction (SPE) was carried out to extract the polar lipids from the lipidic extracts obtained in section 2.3, as described by Márquez-Ruiz et al. (1996), with modifications. Briefly, the lipid extracts were dissolved in 2 mL petroleum ether and loaded onto a Phenomenex Strata SI-1 silica SPE cartridge (500 mg/6 mL), previously conditioned with 10 mL petroleum ether:diethyl ether 90:10 mix (v/v). After the solvent from the loading step passed through the column, the cartridge was washed three times with 5 mL petroleum ether:diethyl ether 90:10 mix. The polar lipid fraction was then eluted and collected with 20 mL diethyl ether, dried using a rotatory evaporator, and reconstituted with 1 mL isopropanol.

The reconstituted polar lipid extracts were then passed through 0.20 μm PVDF syringe filters into HPLC vials and 3 μL were injected into a Dionex UltiMate 3000 series HPLC system (Fisher Scientific, Germering, Germany), using a method based on a previous study (Grüneis et al., 2019). Thus, samples were separated on an Atlantis T3 C18 100 Å column (2.1 mm × 150 mm × 3 μm, Waters, Vienna, Austria), equipped with the corresponding Atlantis T3 C18 precolumn. Mobile phases A and B were acetonitrile/water 60/40 (v/v) and acetonitrile/isopropanol 20/80 (v/v), respectively, both containing 0.1 % formic acid (v/v) and 10 mmol/L ammonium formate. A constant flow rate of 0.3 mL/min was set, and the mobile phase gradient was as follows: 40–100 % B for the first 15 min, 100 % B between minute 15 to minute 25, and 40 % B between minute 25 to minute 27. The oven temperature was 25 °C.

The HPLC system was coupled to a Dual-Pressure Linear Trap-Quadrupole-Orbitrap mass spectrometer (Fisher Scientific), with an ESI source operating in positive mode with the following settings: source voltage 3.5 kV, sheath gas flow 45 AU (N_2_), auxiliary gas flow: 10 AU (N_2_), capillary voltage 25 V, and capillary temperature 300 °C. A mass range of *m/z* 50–2000 was selected. MS/MS analyses were performed in “Top 6” mode, where the six ions with the highest intensity at each time point were selected for fragmentation, with a normalized collision energy of 35 eV. Data analysis was carried out using Xcalibur 4.1.50 (Fisher Scientific), where peak signals were manually integrated from their extracted ion chromatograms (EICs) and compared between samples in terms of area under the curve. Identification of oxidized triacylglycerols was done according to Grüneis et al. (2019).

### 2.12 Untargeted analysis of hydrophilic extracts by HRMS/MS

With the aim of detecting any metabolite of interest that could be associated with the addition of MSG, the hydrophilic extracts from section 2.4 were directly injected into an Agilent G6546 quadrupole-time of flight (Q-TOF) mass spectrometer (Santa Clara, CA, USA). The ESI source operated in positive and negative mode, with the following settings: drying gas flow 10 L/min (N_2_), drying gas temperature 200 °C, sheath gas flow 12 L/min (N_2_), sheath gas temperature 300 °C, nebulizer pressure 35 psi, and capillary voltage 3.5 kV. TOF survey scans included a mass range of *m/z* 50–1700, with a scan time of 250 ms/spectrum. The analyses were performed in Auto MS/MS mode, where the top four ions at each time point were selected for fragmentation, with a normalized collision energy of 20–50 eV.

Data processing was performed with the XCMS and Spectra R packages (Rainer et al., 2022; Smith et al., 2006; Tautenhahn et al., 2008), after conversion of raw data files to mzML format (Chambers et al., 2012). Data import was then followed by smoothing and centroiding by applying a Savitzky-Golay filter. The *centWave*, *peakgroups*, and *peakdensity* algorithms were used for peak detection, retention time alignment, and correspondence analysis, respectively, followed by gap filling. Significantly different features (p < 0.05) between MSG concentrations were filtered and, when unknown features could represent compounds of interest but no fragmentation data was available through Auto MS/MS mode, targeted MS/MS measurement for those features were carried out. Further data analysis was carried out using Agilent Mass Hunter Qualitative Analysis 10.0.

### 2.13 Statistical analysis

GraphPad Prism 9.1.2 (Boston, MA, USA) was used to conduct the pertinent statistical analyses. All experiments were carried out with three independent replicates per group (n = 3). Normal distribution and the presence of significant outliers within groups were evaluated by Shapiro-Wilk and Grubb's tests, respectively, while equal variances between different groups were confirmed via Brown-Forsythe test. Statistically significant differences (p < 0.05) due to MSG addition and storage or cooking were evaluated by two-way analysis of variance (ANOVA). When significant differences were found, the two-way ANOVA was followed by Tukey's post hoc test. Moreover, to further visualize the influence of variables on certain parameters, principal component analysis (PCA) was carried out. In addition, correlations between different parameters were assessed by two-tailed Pearson's correlation test. For chromatographic data, the limits of detection (LOD) and quantification (LOQ) were determined according to a signal-to-noise ratio equal to 3 and 10, respectively (ICH Harmonised Tripartite Guideline, 2005).

## 3 Results and discussion

### 3.1 Evaluation of the potential reactivity of MSG in pork burger patties

To gather an initial understanding on the potential interactions between externally added MSG and the meat, its concentration in the hydrophilic extracts of the burger patties was determined via MS/MS. The presence of glutamate in the extracts of the non-fortified patties could be reported, with a value of 0.10 ± 0.02 mg/patty for the raw meat extracts and values of 0.08 ± 0.01, 0.09 ± 0.01, 0.14 ± 0.01, and 0.08 ± 0.02 mg/patty for the extracts of cooked meat previously stored at 4 °C during 0, 2, 3, and 4 days, respectively. Due to this small amount of free glutamate naturally present in those extracts, results were expressed as a difference between MSG fortification groups, in order to better reflect the amount of supplemented MSG that was recovered during the extraction (Table 1). In all cases, the differences between the groups were much lower than what was initially added to the meat (0.4 % or 1.2 % MSG). This could be due to a limited recovery during the extraction process; however, potential reactions with the meat components could also partially contribute to this glutamate loss. As the amount of glutamate initially added to the 1.2 % MSG group was triple the amount added to the 0.4 % MSG group, a fold-change close to 3 would have been expected to be maintained when comparing the difference between the 0.0 % and 1.2 % MSG groups and the difference between the 0.0 % and 0.4 % MSG groups, despite MSG not being fully recovered in the extraction. Nevertheless, the mean fold-change was below 3 (1.35–2.71) for all raw and cooked meat extracts, suggesting that part of the externally added glutamate was reacting in the meat samples.

**Table 1:** Comparison of the recovered monosodium glutamate concentrations (C_MSG_) and associated ^1^H NMR signal areas (β-CH_2_ and γ-CH_2_) in the hydrophilic extracts of pork meat burger patties.

Storage time – Cooking stage	Recovered C_MSG_ by MS/MS (mg/patty)		Mean fold-change^b^	^1^H NMR Area β-CH_2_ (area units × 10^−3^)		Mean fold-change^b^	^1^H NMR Area γ-CH_2_ (area units × 10^−3^)		Mean fold-change^b^
	Δ_0.0 %–0.4 %_^a^	Δ_0.0 %–1.2 %_^a^		Δ_0.0 %–0.4 %_^a^	Δ_0.0 %–1.2 %_^a^		Δ_0.0 %–0.4 %_^a^	Δ_0.0 %–1.2 %_^a^	
Day 0 – Raw	1.71 ± 1.11	4.63 ± 0.03	2.71	12.26 ± 12.89	31.07 ± 4.74	2.53	20.01 ± 17.10	40.96 ± 5.10	2.05
Day 0 – Cooked	3.17 ± 1.00	4.29 ± 1.33	1.35	29.41 ± 17.68	49.18 ± 9.74	1.67	43.01 ± 23.14	72.44 ± 15.59	1.68
Day 2 – Cooked	4.75 ± 2.37	7.09 ± 0.35	1.49	28.64 ± 12.20	46.05 ± 9.75	1.61	41.71 ± 16.43	63.23 ± 13.36	1.52
Day 3 – Cooked	3.65 ± 0.09	6.72 ± 0.75	1.84	17.34 ± 2.32	34.07 ± 9.69	1.96	23.83 ± 3.80	45.94 ± 13.31	1.93
Day 4 – Cooked	3.93 ± 1.14	9.64 ± 0.40	2.45	23.06 ± 17.37	32.88 ± 8.74	1.43	33.46 ± 25.30	51.65 ± 12.17	1.54

Data is presented as mean ± SD (n = 3). MS/MS: Tandem mass spectrometry; ^1^H NMR: Proton nuclear magnetic resonance.

a Difference between two MSG concentration groups (0.0 %, 0.4 %, or 1.2 % added MSG).

b Fold-change refers to the ratio between Δ_0.0 %–1.2 %_ and Δ_0.0 %–0.4 %_ on the respective parameter measured.

**Fig. 1:**
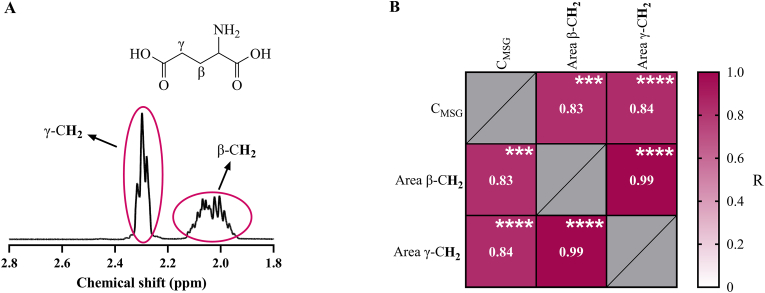
^1^H-NMR signals associated with the methylene groups (CH_2_) of monosodium glutamate (MSG) in positions γ and β (A), and correlation matrix between the concentration of MSG (C_MSG_) obtained by MS analysis and the areas (Area β-CH_2_ and Area γ-CH_2_) obtained by ^1^H-NMR (B). Data in B is presented as Pearson's R coefficients obtained after two-tailed Pearson's correlation test. Statistically significant correlations are indicated with ∗∗∗ (p < 0.001) and ∗∗∗∗ (p < 0.0001).

A similar approach was taken by integrating the areas of the characteristic ^1^H NMR signals of MSG, particularly the signals associated with the protons on the *beta*- and *gamma*-methylene groups, which can be assigned to a multiplet at 2.05 ppm and a triplet at 2.33 ppm, respectively (Fig. 1A). Therefore, the area differences between MSG concentration groups for each set of hydrophilic extracts were calculated and the mean fold-change between them was determined (Table 1). Similar to the MS/MS data, all mean fold-changes comparing these differences had a lower value than 3 for both signals. Specifically, it could be observed that all mean fold-changes related to NMR areas were below 2 for the cooked patties groups, which further hints towards the modification of the externally added glutamate under heating conditions.

A correlation analysis between the results obtained from the two methods was carried out (Fig. 1B), finding a strongly significant positive correlation between the MS/MS data and the ^1^H NMR areas, with Pearson's R coefficients of 0.826 (p < 0.001) and 0.840 (p < 0.0001) in relation to the *beta*-methylene and *gamma*-methylene associated signals, respectively. Naturally, a close relationship was also found between the two types of ^1^H NMR signals (R = 0.987, p < 0.0001). Hence, the combined use of MS/MS and ^1^H NMR for the determination of MSG provides evidence supporting that this additive might undergo modifications or react with compounds in pork patties, especially when they are cooked.

MSG has been suggested to undergo a cyclization and decarboxylation process to form 2-pyrrolidone in heated soybean oil mixtures, as well as to be able to elicit the formation of methylpyrazines when sucrose is simultaneously added (Wu et al., 2000). However, this study used MSG concentration close to 20 % w/w, which clearly exceeds the commonly preferred concentrations for this additive (Bellisle and France, 2008). Furthermore, oils are not among the variety of culinary products that MSG, as a hydrophilic additive, is typically added to (Jinap and Hajeb, 2010; Henry-Unaeze, 2017). Hence, different MSG-related reactions might be at play in other food products. To our knowledge, this is first time that the fate of MSG during the storage and cooking of pork was explored.

### 3.2 Effects of MSG addition on the oxidative stability of pork burger patties

As the determination of glutamate in the pork patties extracts revealed its potential reactivity in the meat, the influence of MSG on the oxidative status of its main macronutrients, namely protein and fats (Vicente and Pereira, 2024), was considered of interest.

#### 3.2.1 Effect on lipid oxidation

**Fig. 2:**
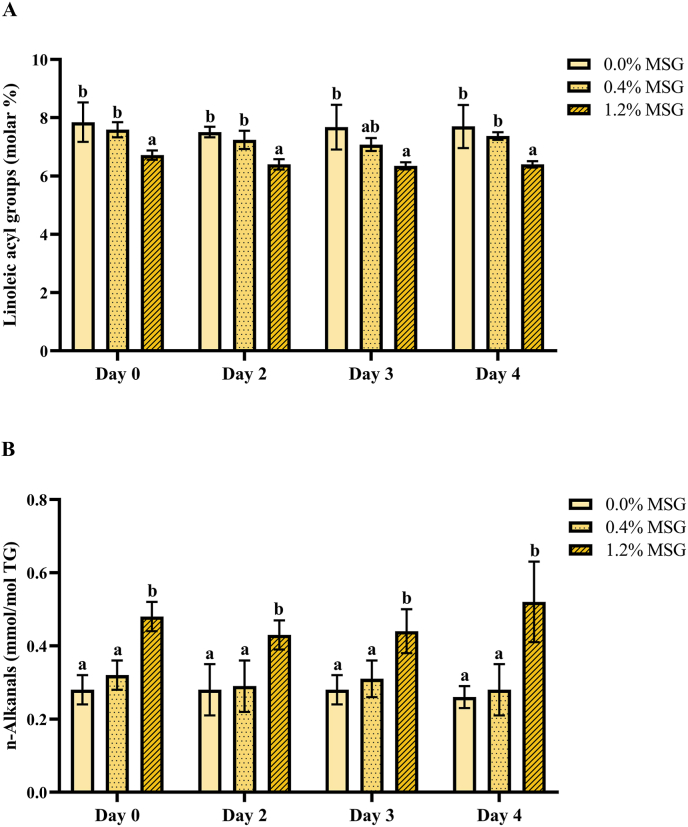
Molar percentage of linoleic acyl groups (A) and concentration of n-alkanals in mmol/mol triacylglycerols (TG) (B) in pork meat burger patties, after different days of storage at 4 °C and cooking at 180 °C. Data is presented as mean ± SD (n = 3). Different lower-case letters (a, b) within the same day of storage indicate statistically significant difference (p < 0.05) between monosodium glutamate (MSG) concentration groups, as determined by two-way analysis of variance (ANOVA) followed by Tukey's post hoc test.

The meat lipid extracts were analyzed by ^1^H NMR and their acyl groups composition was determined (Fig. S1A). Linoleic acyl groups were the most prominent polyunsaturated acyl groups (8 %), and therefore the most likely to show significant changes due to the progress of lipid oxidation. Hence, the influence of MSG supplementation on the molar percentage of linoleic acyl groups was evaluated in the lipidic extracts of the cooked burger patties (Fig. 2A). As shown, fortifying the meat with 0.4 % MSG did not have a statistically significant impact (p > 0.05). However, fortification with 1.2 % MSG showed a significant decrease (p < 0.01) on the content of linoleic acyl groups when comparing it to non-fortified patties, a change that was observed throughout all days of storage. Thus, storage time did not have a significant impact (p > 0.05) on this finding. To find out whether cooking was involved in this effect, the content of linoleic acyl groups was evaluated in a set of raw meat lipidic extracts as well (Fig. S1B). Interestingly, the concentration of MSG did not have a significant effect (p > 0.05) within the raw patties, and cooking the ones with 0.0 % and 0.4 % MSG did not show a significant change either (p > 0.05). Only 1.2 % MSG cooked burger patties displayed a significant decrease (p < 0.05) on linoleic acyl groups, evidencing an interaction effect between MSG supplementation and cooking. Therefore, these results suggest that MSG, when added in high concentrations (1.2 %), promotes the oxidation of linoleic acyl groups when cooking pork burger patties.

To support this evidence, the influence of MSG addition on the formation of lipid oxidation products in the cooked patties was evaluated. In this context, the ^1^H NMR spectral data allowed the detection of n-alkanals, which are a group of common secondary lipid oxidation products (Martínez-Yusta and Guillén, 2014). As displayed in Fig. 2B, the content of n-alkanals was significantly higher (p < 0.05) in the lipidic extracts of 1.2 % MSG cooked burger patties, compared to 0.0 % and 0.4 % MSG patties. As with the linoleic acyl groups, this difference was independent of the storage time of the patties prior to their cooking.

**Fig. 3:**
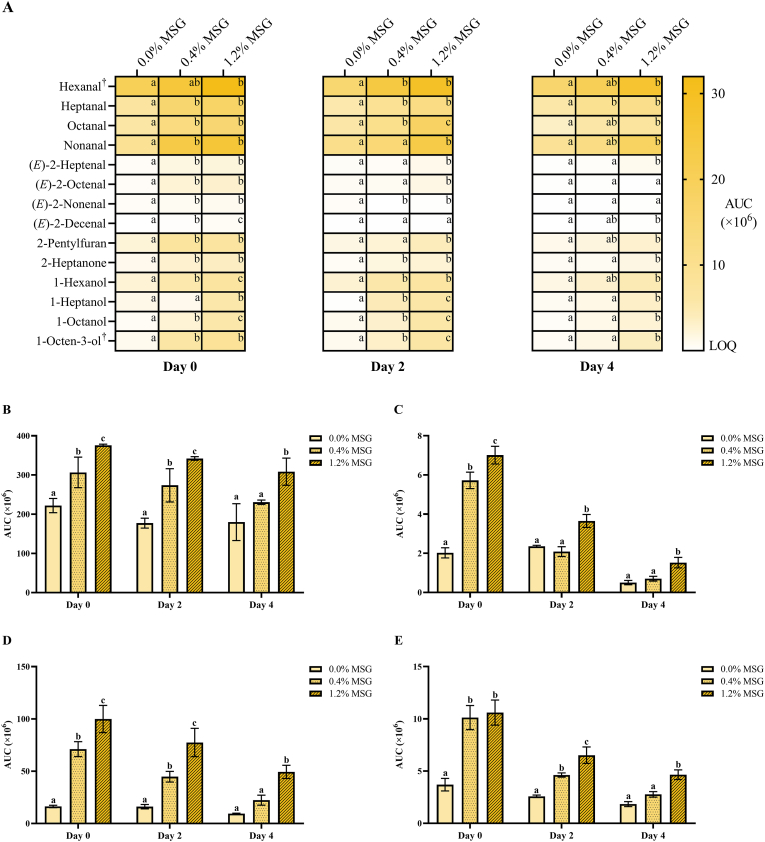
Content of individual volatile oxidation compounds (A), total alkanals (B), total (*E*)-2-alkenals (C), total alcohols (D) and sum of 2-pentylfuran and 2-heptanone (E) in pork meat burger patties, after different days of storage at 4 °C and cooking at 180 °C. Data is presented as mean in A and mean ± SD in B-E (n = 3). Different lower-case letters (a, b, c) within the same day of storage indicate statistically significant difference (p < 0.05) between monosodium glutamate (MSG) concentration groups, as determined by two-way analysis of variance (ANOVA) followed by Tukey's post hoc test. AUC: Area under the curve; LOQ: limit of quantification. †: Data is expressed as area units × 10^−7^ instead of area units × 10^−6^ for visualization purposes.

To gain insights on the specific alkanals formed due to MSG addition, as well as other volatile oxidation compounds (VOCs) that could not be detected by ^1^H NMR, aliquots from the cooked meat were analyzed via SPME/GC-MS (Fig. 3). Samples from day 3 of storage were excluded from this analysis, as no significant effects solely present on that day were expected. Thus, a total of fourteen different VOCs were identified in all meat samples from days 0, 2, and 4 (Fig. 3A), including four alkanals, four (*E*)-2-alkenals, four alcohols, one ketone, and one furan derivative. Among them, hexanal and 1-octen-3-ol were the two most prominent VOCs in all cases, followed by nonanal, octanal, and heptanal, all of which have been described as common volatile compounds in cooked pork by previous studies (Chen et al., 2019; Han et al., 2020). The content of all identified VOCs was significantly higher (p < 0.05) in 1.2 % MSG patties than in the non-fortified ones, with the exception of (*E*)-2-decenal in day 2 samples and (*E*)-2-octenal and (*E*)-2-nonenal in the samples from day 4. The prooxidant effect of 1.2 % MSG falls in line with the ^1^H NMR results described. In addition, GC-MS was able to discriminate a significantly higher content (p < 0.05) for many VOCs in the 0.4 % MSG patties as well, especially in the case of day 0 samples, evidencing that 0.4 % externally added MSG can also promote lipid oxidation to a certain extent.

Alkanals were the major group of VOCs in the meat. Overall, the total alkanals areas, obtained by the combined sum of each individual alkanal (Fig. 3B), clearly displayed the promoting effect of MSG addition on lipid oxidation, while storage did not have a significant influence (p > 0.05) on the results obtained for each concentration group, with the exception of 0.4 % MSG patties of day 4, which had a slight but significantly lower (p < 0.05) alkanals content than 0.4 % MSG patties cooked on day 0. Storage did, however, have a clearer impact on the content of other VOCs. For instance, cooking the meat after a longer storage period, although not explicitly indicated in Fig. 3C, resulted in a significantly lower content of (*E*)-2-alkenals (p < 0.0001). This might be attributed to a slightly more advanced oxidation state in the stored raw meat, as storage is known to promote the oxidation of meat lipids by releasing iron from heme proteins (Domínguez et al., 2019). This advance could have in turn triggered the start of the degradation of certain volatile compounds upon cooking, thereby explaining their lower amount. In this sense, the degradation of (*E*)-2-alkenals in highly oxidized canola oil has been reported before (Grebenteuch et al., 2021). Interestingly, this storage-related reduction caused the prooxidant effect of MSG addition to be less apparent in the cooked patties from days 2 and 4, with the content of certain (*E*)-2-alkenals being statistically similar regardless of the addition of MSG, such as (*E*)-2-octenal and (*E*)-2-nonenal on day 4 samples. Despite this, (*E*)-2-alkenals only constituted a minor fraction of the total meat VOCs. A similar situation could be observed for alcohols, 2-heptanone, and 2-pentylfuran (Fig. 3D and E), in the case of 0.4 % MSG patties. Nevertheless, the prooxidant effect of 1.2 % MSG addition was still clearly significant in all cases (p < 0.001).

Overall, our results provide strong evidence of the stimulating effect that the addition of high levels of MSG has on the formation of VOCs derived from meat lipids during cooking. To test whether this effect additionally applies to non-volatile lipid oxidation products, the polar lipid extracts from the meat were analyzed via LC-HRMS/MS. Only three products corresponding to oxidized triacylglycerols, either with an epoxy or a hydroxyl group, could be identified: TG 54:0 [O]. TG 54:1 [O], and TG 54:3 [O] (Table S3). However, there were no significant differences (p > 0.05) associated with MSG addition on the content of any of them. Moreover, the presence of hydroperoxides among these oxidized triacylglycerols could not be detected. This is relevant, as hydroperoxides are the main primary lipid oxidation products in meat, their absence being indicative of their degradation during cooking as well as the occurrence of termination reactions, suggesting an advanced oxidation stage (Domínguez et al., 2019; Liu et al., 2022).

Hence, it can be inferred that the lipid-related prooxidant effect of MSG during cooking of pork burger patties mainly involves the promotion of secondary oxidation products formation. This is important for consumers and industries, as the generation of these products is associated with off-flavors, which may have a negative impact on the sensory quality, thereby influencing consumers’ acceptability (Amaral et al., 2018). While it is possible that the remaining additional MSG might mask off-flavors to a certain extent, the oxidation of lipids entails a reduction on the nutritional value of meat as well (Domínguez et al., 2019). Furthermore, certain lipid-derived VOCs can be toxic, often associated with pro-inflammatory and mutagenic effects (Sottero et al., 2019).

Administration of MSG has been linked to oxidative kidney damage in rats, with the generation of reactive oxygen species (ROS) via the upregulation of *α*-ketoglutarate dehydrogenase as a plausible mechanism, as well as the inhibition of antioxidant enzymes, such as catalase, glutathione-S-transferase, and superoxide dismutase (Paul et al., 2012; Sharma, 2015). However, it is currently unclear whether this can take place in food. The influence of other additives commonly used in meat products on the progress of lipid oxidation has been investigated by previous studies. Such is the case for sodium chloride, which has been stated to facilitate the oxidation of pork and other meat lipids via different mechanisms, including the release of iron ions, the disruption of the cells membrane integrity, and the inhibition of antioxidant enzymes (Mariutti and Bragagnolo, 2017; Zhao et al., 2020). Chloride ions have been deemed to be the responsible components for this effect rather than the sodium cations, with other chloride salts displaying prooxidant effects as well (Mariutti and Bragagnolo, 2017). Therefore, given the absence of additional chloride ions with MSG supplementation, it is likely that MSG exerts its prooxidant role by a different mechanism than sodium chloride.

Bae and Kim (2024) recently reported the effects of glutamate and MSG addition on the oxidative stability of photosensitized oil-in-water (O/W) emulsions, by analyzing the content of headspace oxygen and conjugated dienes. While they did not directly investigate the impact on secondary lipid oxidation products, their study reported antioxidant or prooxidant effects depending on conditional factors such as the emulsifier used and the presence of light, with MSG generally showing milder effects than glutamate (Bae and Kim, 2024). This illustrates the complexity of MSG interactions with food components. Pork meat is an especially complex food system containing not only lipids as nutrients, but proteins and sugars as well (Vicente and Pereira, 2024; Moreno et al., 2020). Therefore, in order to understand the role of MSG on the oxidation of meat lipids, the potential implication of other molecules present needs to be considered.

#### 3.2.2 Effect on protein oxidation

**Fig. 4:**
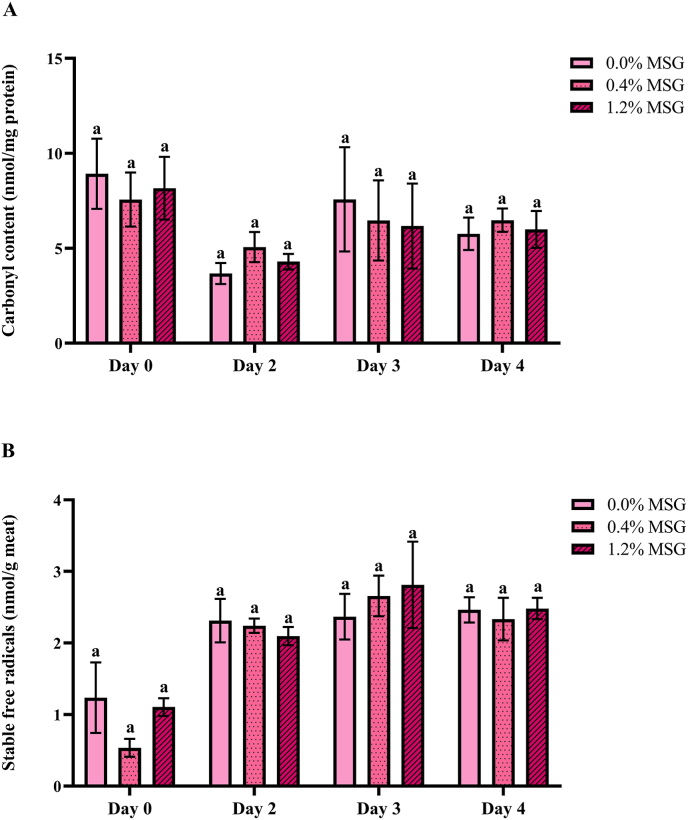
Concentration of protein carbonyls (A) and stable free radicals (B) in pork meat burger patties, after up to 4 days of storage at 4 °C and cooking at 180 °C. Data is presented as mean ± SD (n = 3). An identical lower-case letter (a) within the same storage day indicates no statistically significant difference (p > 0.05) between MSG concentration groups, as determined by two-way analysis of variance (ANOVA).

With pork being a source of proteins, the oxidative state of these biomolecules also affects its quality, as the progress of protein oxidation is associated with protein structure alterations, resulting in lower digestibility and a lower nutritional value, as well as unwanted changes of sensory quality (Domínguez et al., 2021; Lund et al., 2011). Among different products, formation of carbonyls is considered a general marker for assessing damage related to protein oxidation in food (Soglia et al., 2016). Therefore, the content of protein carbonyls in the pork burger patties was analyzed via the DNPH assay. As shown in Fig. 4A, no significant difference due to MSG addition (p > 0.05) was found within any of the cooked patties groups stored for the same amount of days. Storage time did have a slight effect on the protein carbonyls content, albeit only significant (p < 0.05) between samples from day 0 and 2, for which a decrease was observed. Nevertheless, no significant differences (p > 0.05) between the patties cooked at the beginning of the storage period and the patties cooked at the end were discovered, suggesting that storage of pork patties at 4 °C up to 4 days does not increase their carbonyl content. Conversely, cooking resulted in a significant increase (p < 0.01) on protein carbonyls for burger patties with 0.0 % and 0.4 % additional MSG (Fig. S2A). In the case of 1.2 % MSG patties, no significant change on these carbonyls was observed due to the cooking process (p > 0.05), as the raw patties with 1.2 % MSG had a seemingly higher mean value than the other raw patties. However, this difference was not statistically significant (p > 0.05), therefore the protein prooxidant role for this concentration of MSG could not be demonstrated in raw patties as well. Overall, cooking of the patties was the main factor promoting protein carbonyls formation, with any possible contribution from MSG addition being negligible.

Heat treatments are known to have an impact on protein oxidation, since they can cause the denaturation of proteins such as myoglobin, releasing heme iron and, in turn, producing free radicals via protein complexation (Domínguez et al., 2021). The carbonyl content reached after cooking was comparable to processed pork products such as dry-cured sausages (Armenteros et al., 2009). In this sense, previous studies have considered both cooking and processing as main critical factors on the oxidation of proteins from different meat products, while having a milder impact on lipid oxidation (Domínguez et al., 2021; Goethals et al., 2020). This is in agreement with our study, since a significant decrease on the content of linoleic acyl groups during cooking was only found with the help of MSG supplementation (Fig. S1B), whereas the increase of protein carbonyls took place in the non-fortified patties as well.

To find out whether cooking, storage time or MSG addition had an effect on the content of stable free radicals, and therefore relate those effects to protein or lipid oxidation, freeze-dried aliquots of the burger patties were analyzed by ESR. As shown in Fig. 4B, the concentration of stable free radicals was not significantly influenced by the addition of MSG (p > 0.05). However, a significant difference (p < 0.01) was observed between the samples cooked after 0 days of storage and the samples cooked on the rest of days, indicating that the content of stable free radicals in the meat increased between day 0 and day 2. Likewise, MSG addition had no significant impact (p > 0.05) in the raw patties (Fig. S2B), whereas cooking caused a clear significant decrease (p < 0.01). The factors that caused significant changes on the content of these radicals were the same that had an impact on protein oxidation as well. Thus, the decrease on the stable free radicals content during cooking, as indicated by the unpaired electrons, may be due to their reaction with proteins, thereby explaining the simultaneous increase in protein carbonyls. A significant negative correlation (R = −0.6694, p < 0.01) was found between the stable free radical and protein carbonyl contents, supporting this explanation and highlighting them, within the context of this study, as a reflection of each other. Nevertheless, it should be pointed out that short-lived radicals may have also had an impact on the extent of protein oxidation, as their high reactivity makes them prone to participate in this process as well (Lund et al., 2011).

Protein and lipid oxidation have been previously described as interrelated processes that can take place simultaneously during meat heat treatments (Traore et al., 2012; Wen et al., 2019). However, it has been also pointed out that the reactions involved for protein oxidation are kinetically slower compared to lipid oxidation (Hadidi et al., 2022), which may explain the absence of a significant impact on protein carbonyls despite the increased amount of lipid oxidation products. Therefore, future research should evaluate whether cooking of MSG-fortified patties under other conditions, such as longer time or higher temperature, could additionally result in the promotion of protein oxidation.

### 3.3 Occurrence of the Maillard reaction in MSG-fortified pork burger patties

The Maillard reaction is a valuable process for the food industry as it is responsible for the formation of different desirable flavors in food products (Starowicz and Zieliński, 2019). Nevertheless, it can also be a matter of concern, since certain MRPs, such as advanced glycation end products (AGEs), are associated with the promotion of health issues including cardiovascular diseases, kidney damage, diabetes, and neurological disorders (Golchinfar et al., 2023). Existing evidence suggests that MSG can influence flavors in food by participating in the Maillard reaction due to its free amino group (Wu et al., 2000; Wijayasekara and Wansapala, 2017), although the mechanism and intermediary compounds by which this effect can take place are not fully understood. Given MSG's reactivity as well as existing links between the Maillard reaction and lipid oxidation (Kou et al., 2024), it is reasonable to hypothesize that the role of MSG in the pork patties might involve the promotion of the Maillard reaction.

**Fig. 5:**
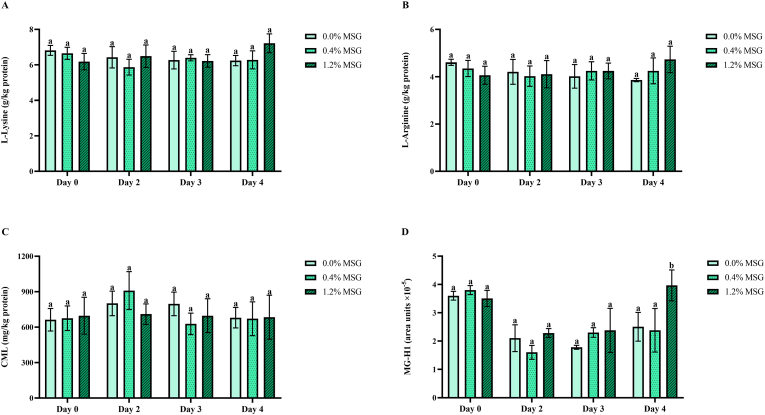
Concentration of protein-bound L-lysine (A), L-arginine (B), and CML (C) in pork meat burger patties after up to 4 days of storage at 4 °C and cooking at 180 °C, as well as their area values for methylglyoxal-derived hydroimidazolone 1 (D). Data is presented as mean ± SD (n = 3). Different lower-case letters (a, b) within the same storage day indicate statistically significant difference (p < 0.05) between monosodium glutamate (MSG) concentration groups as determined by two-way analysis of variance (ANOVA) followed by, in case of a significant effect detected, Tukey's post hoc test. AUC: Area under the curve; CML: N(6)-carboxymethyllysine.

Hence, the amino acid precursors for a series of AGEs, which are late-stage MRPs, were analyzed in the cooked meat samples. As shown in Fig. 5, MSG addition did not have a significant impact (p > 0.05) on the concentration of protein-bound lysine (Fig. 5A) and arginine (Fig. 5B) for any of the storage days on which the burger patties were cooked. Storage time did not have a significant effect (p > 0.05) on the concentration of these two amino acids either. This suggests that the introduction of modifications on these two amino acids does not take part as one of the effects of MSG on pork, regardless of the time stored at 4 °C. In order to corroborate this, specific AGEs derived from them were analyzed. Since CML is the most commonly used AGEs marker (Hull et al., 2012), it was chosen as the main representative for lysine-derived AGEs and therefore quantified (Fig. 5C). Its concentration in the cooked patties (around 700 mg/kg) was comparable to some commercial meat products made out of beef and pork, which showed high amounts of CML after cooking (Yu et al., 2022). Nevertheless, neither the addition of MSG nor the time of storage significantly influenced (p > 0.05) the concentration of this AGE. The same situation was observed for the rest of lysine-derived AGEs analyzed, namely N(6)-carboxyethyllysine (CEL), glyoxal-lysine dimer (GOLD), and methylglyoxal-lysine dimer (MOLD), where storage and MSG supplementation did not cause any significant change on their area under the curve values (Table S4). Regarding arginine-derived AGEs, glyoxal-derived hydroimidazolone 1 (G-H1) did not show any significant change on its content (p > 0.05) due to MSG addition or storage. The same was true for pentosidine, derived from both lysine and arginine. However, the addition of 1.2 % MSG resulted in a significantly increased methylglyoxal-derived hydroimidazolone 1 (MG-H1) content (p < 0.001) in the patties cooked after 4 days of storage (Fig. 5D). MG-H1 is formed from the reaction between arginine and the α-dicarbonyl methylglyoxal, the latter of which can originate from lipid oxidation (Lin et al., 2023). Lin et al. (2023) previously highlighted MG-H1 as one of the most prominent AGE in canned meat and seafood, and described a positive correlation between its content and the formation of methylglyoxal. Therefore, it is likely that the prooxidant effect of MSG resulted in the increased formation of methylglyoxal, promoting the generation of MG-H1. Despite this, it can be inferred that these variations on the content of MG-H1 were rather minimal, as they were not enough to significantly alter the concentration of arginine (Fig. 5B).

**Fig. 6:**
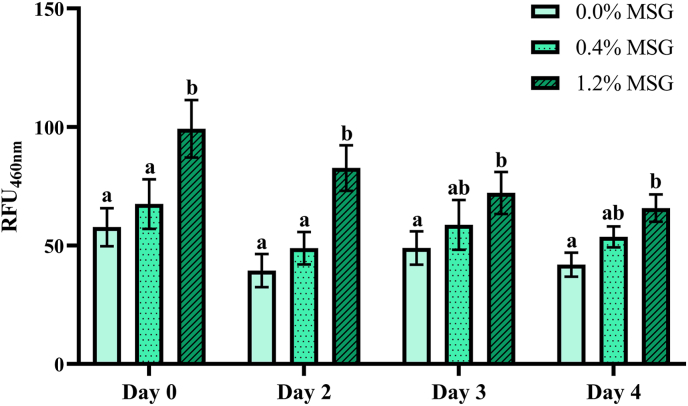
Schiff bases content in pork meat burger patties after storage at 4 °C for up to 4 days and cooking at 180 °C. Different lower-case letters (a, b) within the same day of storage indicate statistically significant difference (p < 0.05) between monosodium glutamate (MSG) concentration groups, as determined by two-way analysis of variance (ANOVA) followed by Tukey's post hoc test. RFU: relative fluorescence units.

Since AGEs formation was not one of main effects of MSG addition, its influence on the content of early-stage MRPs, namely Schiff bases, was evaluated (Fig. 6). As shown, fortification of the patties with 1.2 % MSG significantly increased (p < 0.01) the relative fluorescence emission at 460 nm in the pork samples, indicating the formation of this type of compounds. This effect was present throughout the entire storage period. However, similar to some of the VOCs detected by GC-MS, the impact was less pronounced when the storage time until cooking was longer.

The determination of Schiff bases is often used as a marker of protein oxidation in meat, as they can be formed through the reaction between protein carbonyls, such as α-aminoadipic semialdehyde (AAS), and the ε-amino group in protein-bound lysine or arginine residues (Armenteros et al., 2009; Utrera et al., 2012). However, lipid-derived carbonyls, as well as carbonyl compounds of other origin, may also cause the formation of Schiff base structures (Gatellier et al., 2009). Considering that both the protein carbonyls content and the concentration of protein-bound lysine and arginine were not influenced by MSG supplementation, it is unlikely for the additional Schiff bases formation to have taken place via the AAS pathway. Instead, since MSG was supplemented in free glutamate form, it is highly possible that the Schiff bases detected were formed via the reaction between the α-amino group of MSG and carbonyl groups from secondary lipid oxidation products or reducing sugars.

**Fig. 7:**
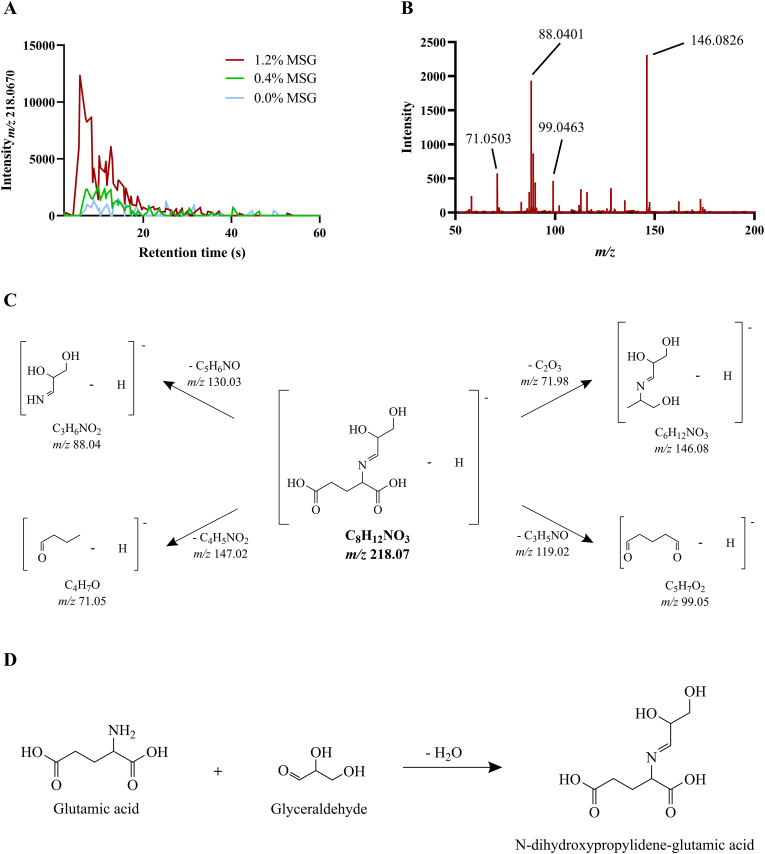
Exemplary extracted ion chromatogram at *m/z* 218.0670 for a set of pork meat burger patties hydrophilic extracts with different monosodium glutamate (MSG) concentration (A), as well as MS2 spectrum (B), proposed fragmentation pathway (C), and proposed formation reaction (D) for the Schiff base tentatively identified as N-dihydroxypropylidene-glutamic acid.

In order to uncover the identity of the specific Schiff bases responsible for the effect reported in Fig. 6, HRMS/MS was utilized. The analysis of the polar lipid extracts could not reveal the presence of any compound that could be directly attributed to MSG addition. On the other hand, the analysis of the hydrophilic meat extracts disclosed an increase in the signal of a feature with a *m/z* value of 218.0670, especially marked in cooked meat patties supplemented with 1.2 % MSG (Fig. 7A). After obtaining the MS/MS spectrum associated with this feature (Fig. 7B), its identity was assigned to the [M-H]^-^ adduct of N-dihydroxypropylideneglutamic acid, with a mass error of 0.24 ppm and its proposed fragmentation pathway shown in Fig. 7C. The formation of this Schiff base could be attributed to the carbonyl-amine reaction between glutamic acid, externally added via MSG supplementation, and glyceraldehyde (Fig. 7D). As shown in Table 2, the peak areas of its extracted ion chromatograms were significantly higher (p < 0.05) in the 1.2 % MSG patties group compared to the 0.0 % MSG group regardless of storage time. Interestingly, for the 0.0 % MSG group, the presence of N-dihydroxypropylideneglutamic acid could be detected when the patties were cooked on day 0 of storage, whereas its amount on the other days was below the limit of detection, suggesting that a base amount of this Schiff base could be naturally present in the pork patties and that storage might hinder its formation. Fittingly, N-dihydroxypropylideneglutamic acid was detected in all 0.4 % MSG patties, although its peak area in patties cooked on day 0 was significantly higher (p < 0.05) than in the patties cooked on the rest of the storage days. Conversely, storage time did not seem to have a significant impact (p > 0.05) on the signal of this Schiff base in 1.2 % MSG patties.

**Table 2:** Areas under the curve (AUC) indicating the content of N-dihydroxypropylidene-glutamic acid in pork meat burger patties, after up to 4 days of storage at 4 °C and cooking at 180 °C.

MSG concentration (%)	AUC_*m/z*__218.07_ (area units × 10^−4^)			
	Day 0	Day 2	Day 3	Day 4
0.0	3.28 ± 0.35^a^	< LOD	< LOD	< LOD
0.4	4.74 ± 0.82^ab^	2.59 ± 1.46^a^	2.58 ± 0.68^a^	2.35 ± 0.98^a^
1.2	5.08 ± 0.49^b^	3.34 ± 1.37^a^	3.58 ± 1.06^a^	5.14 ± 1.09^b^

Data is presented mean ± SD (n = 3). Different superscript lower-case letters (a, b) within the same column (storage day) indicate statistically significant difference (p < 0.05) between monosodium glutamate (MSG) concentration groups, as determined by two-way analysis of variance (ANOVA) followed by Tukey's post hoc test. LOD: Limit of detection.

Glyceraldehyde has been stated to form MRPs by previous research (Kitahara et al., 2008). Its availability for reaction might be related to the thermal degradation of glycolytic intermediates released during *postmortem* metabolism (Hamilton et al., 2003; Usui et al., 2007). In this sense, peptide markers for glyceraldehyde-3-phosphate dehydrogenase have been identified in meat before (Montowska and Spychaj, 2018), which suggests the presence of these intermediate metabolites. It can be hypothesized that, after a longer storage time, *postmortem* metabolism may be more advanced, leaving a lower amount of intermediary compounds available, which could in turn explain the seemingly less pronounced formation of the Schiff base. Alternatively, it is unclear whether MSG can react with other meat components during storage, hindering the formation of Schiff bases in favor of other reactions during cooking. Future studies should investigate these hypotheses, in order to provide full understanding on the origin of N-dihydroxypropylideneglutamic acid in pork.

**Fig. 8:**
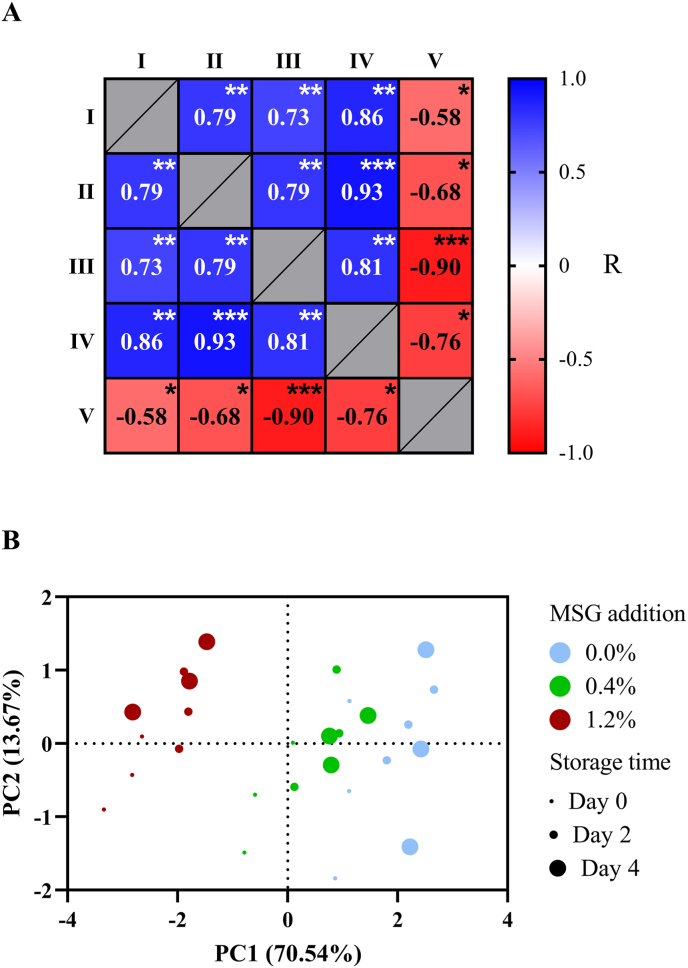
Correlation matrix (A) and principal component score plot (B) in relation to the content of N-dihydroxypropylidene-glutamic acid (I), Schiff bases (II), n-alkanals determined by ^1^H-NMR (III), n-alkanals determined by SPME/GC-MS (IV), and linoleic acyl groups (V). Data in A is presented as Pearson's R coefficients obtained after two-tailed Pearson's correlation test. Statistically significant correlations are indicated with ∗ (p < 0.05), ∗∗ (p < 0.01), ∗∗∗ (p < 0.001), and ∗∗∗∗ (p < 0.0001). PC: principal component.

Given the existing relationship between the Maillard reaction and lipid oxidation, the involvement of this Schiff base on the prooxidant effect of MSG seems plausible. Hence, a correlation analysis was carried out (Fig. 8A), considering the content of N-dihydroxypropylideneglutamic acid, Schiff bases, linoleic acyl groups, and alkanals, the latter being selected as the main representative secondary lipid oxidation products identified in the meat patties. Indeed, a significant correlation (p < 0.05) was found between all of these parameters, suggesting a link between Schiff bases formation and the promotion of lipid oxidation during cooking of the burger patties. While a certain stability of N-dihydroxypropylideneglutamic acid as a Schiff base is evidenced by its positive correlation with the Schiff bases fluorometric measurement (R = 0.787, p < 0.01), its partial rearrangement into an Amadori product cannot be ruled out, since Schiff bases are prone to go through this isomerization reaction (Dean et al., 1997). Previous research suggests that Amadori products are able to promote lipid oxidation via the generation of ROS, such as superoxide radicals (Oak et al., 2000; Zamora and Hidalgo, 2011). Consequently, N-dihydroxypropylideneglutamic acid might exert a prooxidant effect in a similar manner, following Amadori rearrangement. Nevertheless, further studies should evaluate the progress of lipid oxidation when adding this Schiff base to simple models and meat products, in order to confirm this mechanism.

Overall, it can be affirmed that MSG supplementation was the main factor influencing the reported results for the cooked burger patties, as supported by the PCA score plot shown in Fig. 8B. As displayed, three clusters, each of them belonging to a different MSG concentration used, can be differentiated, with the 1.2 % MSG cluster especially separated from other two. In addition, the replicates with 0 days of storage show a slight separation from the rest of the samples within the corresponding MSG concentration cluster, evidencing certain influence from storage, as indicated throughout this section.

Thus, our findings show that adding 1.2 % MSG to pork meat burger patties can lead to a poorer quality, as it may indirectly promote the formation of toxic lipid oxidation compounds via the Maillard reaction. In the European Union (EU), a limit of 1 % glutamic acid in food products (w/w), for MSG and other glutamate forms, was previously established by the European Commission (Regulation, 2008). The concentration added to the 1.2 % MSG group, equivalent to 1.05 % glutamic acid (w/w), while chosen based on consumer preferences, was close to this limit. Adding the fact that there is no standing maximum limit outside the EU, this raises the question of whether the addition of MSG, in commonly used concentrations, could potentially compromise the quality of the food products and represent a safety concern in a long-term basis. Hence, further studies should evaluate the extent of the prooxidant effect of different MSG concentrations with a quantitative approach, as this might point towards the need of re-assessing the limit of MSG that can be considered safe. In addition, the effect of other glutamate salts should be similarly evaluated as well, since their effects might differ depending on the glutamate form used (Bae and Kim, 2024), which the current regulations do not distinguish in terms of maximum limit.

## 4 Conclusion

Supplementation of pork burger patties with MSG, using high levels within common consumer preferences, promoted the progress of lipid oxidation during cooking at 180 °C for 15 min, after a storage period at 4 °C up to 4 days. This led to the increased generation of multiple volatile secondary lipid oxidation products, some of them known to be toxic or associated with health detrimental effects. This finding was positively correlated with the formation of Schiff bases such as N-dihydroxypropylideneglutamic acid, via the participation of glutamate in the Maillard reaction. Our results suggest a link between the formation of glutamate-derived early-stage MRPs and the progress of lipid oxidation, possibly via Amadori rearrangement and subsequent ROS generation, which could raise concerns regarding the continuous use of MSG as an additive, in terms of food quality and safety. Therefore, our work described, for the first time, the simultaneous occurrence of Schiff bases structures and secondary lipid oxidation products due to MSG supplementation, providing important insights on the reactivity of this additive in pork during cooking.

## CRediT authorship contribution statement

**Arturo Auñon-Lopez:** Conceptualization, Formal analysis, Investigation, Methodology, Visualization, Writing – original draft, Writing – review & editing. **Verena Rohringer:** Formal analysis, Investigation, Visualization, Writing – review & editing. **Kübra Taranaci:** Formal analysis, Investigation, Writing – review & editing. **Jon Alberdi-Cedeño:** Conceptualization, Formal analysis, Investigation, Methodology, Supervision, Writing – review & editing. **Marc Pignitter:** Conceptualization, Funding acquisition, Methodology, Project administration, Resources, Supervision, Writing – review & editing.

## Declaration of competing interest

The authors declare that they have no known competing financial interests or personal relationships that could have appeared to influence the work reported in this paper.

## Data Availability

Data will be made available on request.
