# Supplementary material for: Addition of monosodium glutamate can reduce the oxidative stability of lipids in pork burger patties via early-stage Maillard reaction products formation
Source: Curr Res Food Sci. 2025 May 20;10:101091. doi: 10.1016/j.crfs.2025.101091 (PMC12152919; doi:10.1016/j.crfs.2025.101091)
Supplement: Multimedia component 1 [file mmc1.docx]

**SUPPLEMENTARY MATERIAL**

**Addition of monosodium glutamate can reduce the oxidative stability of lipids in pork meat burger patties via early-stage Maillard reaction products formation**

Arturo Auñon-Lopez^a,b,#^, Verena Rohringer^a,#^, Kübra Taranaci^a^, Jon Alberdi-Cedeño^a,c,*^
Marc Pignitter^a,*^

^a^Institute of Physiological Chemistry, Faculty of Chemistry, University of Vienna, Josef-Holaubek-Platz 2, 1090 Vienna, Austria

^b^Vienna Doctoral School in Chemistry (DoSChem), Faculty of Chemistry, University of Vienna, Währinger Str. 42, 1090 Vienna, Austria

^c^Food Technology, Faculty of Pharmacy, Lascaray Research Center, University of the Basque Country (UPV-EHU), Paseo de la Universidad nº 7, 01006 Vitoria-Gasteiz, Spain

#Equal contribution

*Corresponding authors: [jon.alberdi@ehu.eus](mailto:jon.alberdi@ehu.eus) (J. Alberdi-Cedeño); [marc.pignitter@univie.ac.at](mailto:marc.pignitter@univie.ac.at) (M. Pignitter)

**Table S1.** Multiple reaction monitoring (MRM) mode transitions used for the determination of glutamate, advanced glycation end products, L-lysine, and L-arginine.

| Analyte | Precursor ion (*m/z*) | Product ions | | | | Source |
| --- | --- | --- | --- | --- | --- | --- |
|  |  | Quantifier (*m/z*) | CE (V) | Qualifier (*m/z*) | CE (V) |  |
| MSG | 189.0 | 84.0 | -20 | 56.0 | -20 | † |
| CML | 205.0 | 84.0 | -19 | 130.0 | -14 | [1] |
| CEL | 219.0 | 84.0 | -18 | 130.0 | -12 | [1] |
| MOLD | 341.3 | 84.0 | -42 | - | - | [2] |
| GOLD | 327.7 | 84.0 | -34 | - | - | [2] |
| MG-H1 | 229.0 | 114.0 | -30 | 211.0 | -10 | [2,3] |
|  |  |  |  | 166.0 | -20 |  |
|  |  |  |  | 70.0 | -20 |  |
| G-H1 | 215.2 | 70.0 | -30 | - | - | [2] |
| Pentosidine | 379.0 | 250.0 | -22 | 187.0 | -30 | [1] |
| L-Lysine | 147.0 | 130.0 | -10 | 84.0 | -20 | [4,5] |
| L-Arginine | 175.0 | 116.0 | -20 | 70.0 | -30 | [4-6] |
|  |  |  |  | 60.0 | -30 |  |

CE: collision energy; CML: N(6)-carboxymethyllysine; CEL: N(6)-carboxyethyllysine; MOLD: methylglyoxal-lysine dimer; GOLD: glyoxal-lysine dimer; MG-H1; methylglyoxal-derived hydroimidazolone 1; G-H1: glyoxal-derived hydroimidazolone 1.

†: Transition confirmed with standard.

**Table S2.** Standard curves and quantification parameters used in the present study

| Analyte | Curve equation | R^2^ | Range | LOD | LOQ |
| --- | --- | --- | --- | --- | --- |
| MSG | y = 169664·x + 4006622 | 0.9926 | 5.0-1000.0 µmol/L | 0.2 µmol/L | 0.5 µmol/L |
| CML | y = 331722·x + 1782 | 0.9994 | 0.5-50.0 mg/L | 0.2 mg/L | 0.4 mg/L |
| L-Lysine | y = 198127·x + 3231251 | 0.9988 | 50.0-500.0 mg/L | 0.2 mg/L | 0.8 mg/L |
| L-Arginine | y = 436562·x + 37074216 | 0.9602 | 50.0-500.0 mg/L | 0.3 mg/L | 0.5 mg/L |

LOD: limit of detection; LOQ; limit of quantification; MSG: monosodium glutamate; CML: N(6)-carboxymethyllysine.

**Table S3.** Oxidized acyltriglycerols identified in polar lipid extracts of pork meat burger patties, based on Grüneis et al. [7].

| Oxidized TG | Molecular formula | Measured precursor mass (*m/z*) | Theoretical mass (*m/z*) | Mass error (ppm) | Mass adduct | Product masses (*m/z*) |
| --- | --- | --- | --- | --- | --- | --- |
| TG 18:0/18:0/18:0 [O] | C_57_H_108_O_7_ | 922.841 | 922.844 | 3.28 | [M+NH_4_]^+^ | 621.541 |
|  |  |  |  |  |  | 603.536 |
| TG 18:0/18:0/18:1 [O] | C_57_H_106_O_7_ | 920.826 | 920.829 | 3.34 | [M+NH_4_]^+^ | 621.540 |
|  |  |  |  |  |  | 603.537 |
|  |  |  |  |  |  | 601.522 |
| TG 18:1/18:1/18:1 [O] | C_57_H_102_O_7_ | 916.794 | 916.797 | 4.01 | [M+NH_4_]^+^ | 631.493 |
|  |  |  |  |  |  | 617.516 |
|  |  |  |  |  |  | 603.537 |

TG: triacylglycerol.

**Table S4.** Areas of different advanced glycation end products (AGEs) found in pork meat burger patties after up to 4 days of storage at 4 °C and cooking at 180 °C.

| Storage day | MSG concentration (%) | Area under curve (area units × 10^-5^) | | | | |
| --- | --- | --- | --- | --- | --- | --- |
|  |  | CEL  (Area*_m/z_* _219 > 84_) | MOLD  (Area*_m/z_* _341 > 84_) | GOLD  (Area*_m/z_* _327 > 84_) | G-H1  (Area*_m/z_* _215 > 70_) | Pentosidine  (Area*_m/z_* _379 > 250_) |
| Day 0 | 0.0 | 90.64 ± 19.77^a^ | 1.53 ± 0.06^a^ | 1.51 ± 0.16^a^ | 5.82 ± 0.66^a^ | 0.42 ± 0.05^a^ |
|  | 0.4 | 97.98 ± 15.20^a^ | 1.85 ± 0.54^a^ | 2.15 ± 0.63^a^ | 7.64 ± 2.58^a^ | 0.45 ± 0.11^a^ |
|  | 1.2 | 116.21 ± 4.88^a^ | 3.25 ± 1.22^a^ | 3.75 ± 1.60^a^ | 11.32 ± 2.63^a^ | 0.50 ± 0.05^a^ |
| Day 2 | 0.0 | 109.20 ± 20.82^a^ | 2.88 ± 1.29^a^ | 3.32 ± 1.76^a^ | 8.69 ± 2.35^a^ | 0.49 ± 0.16^a^ |
|  | 0.4 | 103.80 ± 14.10^a^ | 2.32 ± 1.79^a^ | 2.63 ± 2.50^a^ | 8.24 ± 2.89^a^ | 0.46 ± 0.13^a^ |
|  | 1.2 | 105.42 ± 8.73^a^ | 2.29 ± 0.75^a^ | 2.39 ± 0.96^a^ | 7.39 ± 2.30^a^ | 0.43 ± 0.04^a^ |
| Day 3 | 0.0 | 105.74 ± 7.98^a^ | 2.58 ± 1.38^a^ | 3.16 ± 1.98^a^ | 7.19 ± 2.09^a^ | 0.51 ± 0.11^a^ |
|  | 0.4 | 110.73 ± 26.00^a^ | 2.53 ± 1.24^a^ | 2.95 ± 1.75^a^ | 8.09 ± 3.40^a^ | 0.46 ± 0.11^a^ |
|  | 1.2 | 103.63 ± 8.20^a^ | 2.13 ± 0.90^a^ | 2.40 ± 1.19^a^ | 7.88 ± 1.82^a^ | 0.47 ± 0.05^a^ |
| Day 4 | 0.0 | 116.14 ± 2.41^a^ | 2.93 ± 0.23^a^ | 3.35 ± 0.19^a^ | 8.93 ± 1.92^a^ | 0.53 ± 0.02^a^ |
|  | 0.4 | 98.89 ± 0.97^a^ | 2.60 ± 1.09^a^ | 2.77 ± 1.52^a^ | 9.82 ± 1.66^a^ | 0.43 ± 0.09^a^ |
|  | 1.2 | 101.31 ± 22.32^a^ | 1.94 ± 0.69^a^ | 2.17 ± 1.14^a^ | 8.16 ± 1.29^a^ | 0.43 ± 0.10^a^ |

Data is presented as mean ± SD (n = 3). An identical lower-case letter (a) within the same storage day indicates no statistically significant difference (p > 0.05) between MSG concentration groups for a compound, as determined by two-way analysis of variance (ANOVA). MSG: monosodium glutamate; CEL: N(6)-carboxyethyllysine; MOLD: methylglyoxal-lysine dimer; GOLD: glyoxal-lysine dimer; G-H1: glyoxal-derived hydroimidazolone 1.

**
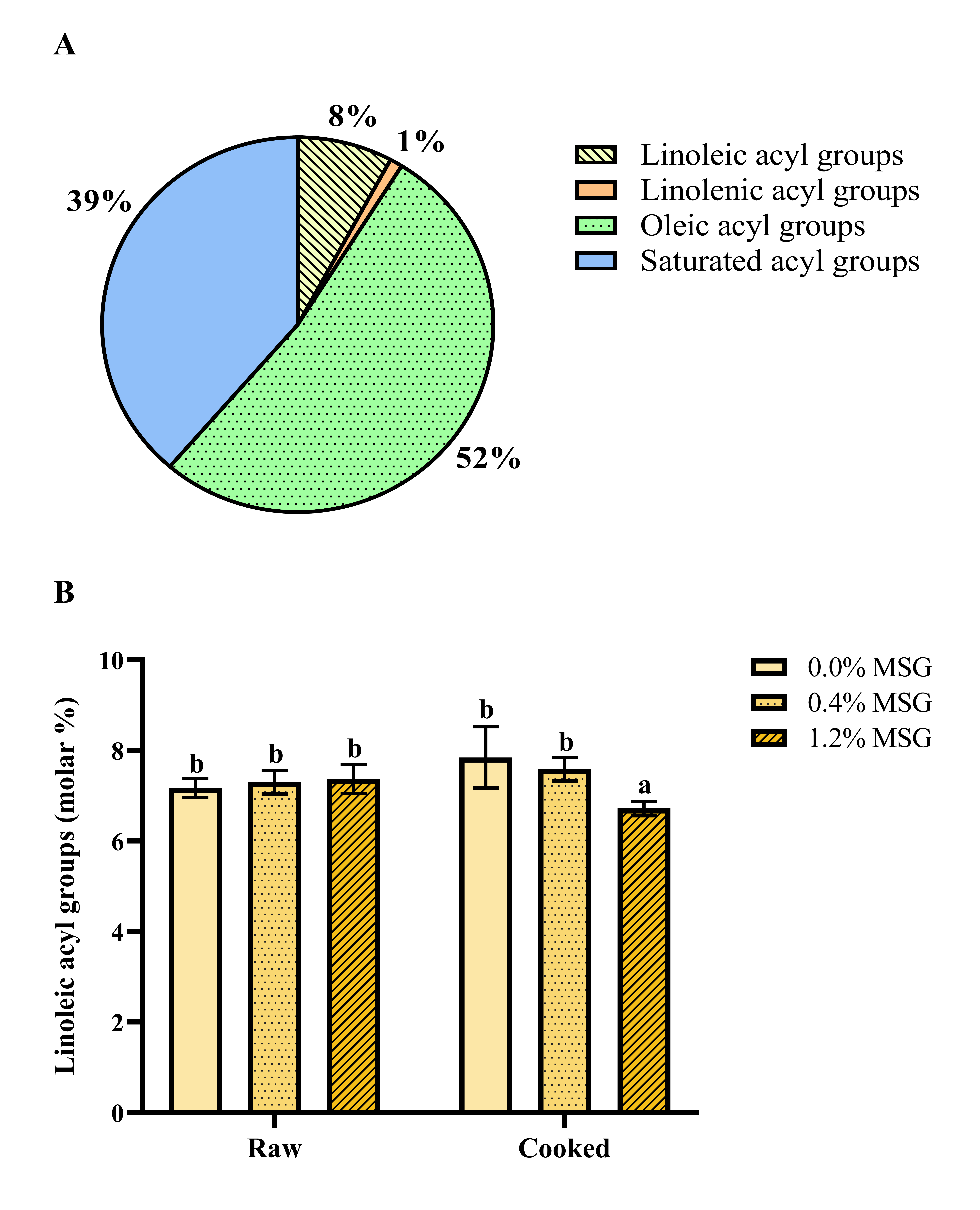
**

**Fig. S1.** Acyl groups composition of the pork meat burger patties at day 0 of storage (A) and their content of linoleic acyl groups before and after cooking at 180 °C for 15 min (B). Data is presented as mean in A and as mean ± SD in B (n ≥ 3). Different lower-case letters in B (a, b) within a cooking stage indicate statistically significant difference (p < 0.05), as determined by two-way analysis of variance (ANOVA) followed by Tukey’s post hoc test.

**

**

**Fig. S2.** Concentration of protein carbonyls (A) and unpaired electrons (B) in pork meat burger patties at day 0 of storage, prior and after cooking at 180 °C for 15 min. Data is presented as mean ± SD (n = 3). An identical lower-case letter (a) within the same cooking stage indicates no statistically significant difference (p > 0.05) between monosodium glutamate (MSG) concentration groups, while an asterisk indicate statistically significant difference (p < 0.001) due to cooking, as determined by two-way analysis of variance (ANOVA) followed by Tukey’s post hoc test.

**References**

[1] G. Zhang, G. Huang, L. Xiao, A.E. Mitchell, Determination of advanced glycation endproducts by LC-MS/MS in raw and roasted almonds (*Prunus dulcis*), J Agric Food Chem. 59 (2011) 12037-12046. <https://doi.org/10.1021/jf202515k>.

[2] Y.Y. Lin, S.F. Huang, K.W. Liao, C.T. Ho, W.L. Hung, Quantitation of α-dicarbonyls, lysine- and arginine-derived advanced glycation end products, in commercial canned meat and seafood products, J. Agric. Food Chem. 71 (2023) 6727-6737. <https://doi.org/10.1021/acs.jafc.3c01205>.

[3] E. Baye, A.B. Mark, M.W. Poulsen, J.M. Andersen, L.O. Dragsted, S.G. Bügel, B. de Courten, 2019. Associations between urinary advanced glycation end products and cardiometabolic parameters in metabolically healthy obese women. J. Clin. Med. 8, 1008. <https://doi.org/10.3390/jcm8071008>.

[4] J. Gómez-Ariza, M. Villegas-Portero, V. Bernal-Daza, Characterization and analysis of amino acids in orange juice by HPLC–MS/MS for authenticity assessment, Anal. Chim. Acta. 540 (2005) 221-230. <https://doi.org/10.1016/j.aca.2004.08.048>.

[5] Z. Liu, M.J. Tu, C. Zhang, J.L. Jilek, Q.Y. Zhang, A.M. Yu, A reliable LC-MS/MS method for the quantification of natural amino acids in mouse plasma: Method validation and application to a study on amino acid dynamics during hepatocellular carcinoma progression, J. Chromatogr., B. 1124 (2019) 72-81. <https://doi.org/10.1016/j.jchromb.2019.05.039>.

[6] J. Martens-Lobenhoffer, S.M. Bode-Böger, Quantification of L-arginine, asymmetric dimethylarginine and symmetric dimethylarginine in human plasma: a step improvement in precision by stable isotope dilution mass spectrometry, J. Chromatogr., B. 904 (2012) 140-143. <https://doi.org/10.1016/j.jchromb.2012.07.021>.

[7] V. Grüneis, S. Fruehwirth, M. Zehl, J. Ortner, A. Schamann, J. König, M. Pignitter, Simultaneous analysis of epoxidized and hydroperoxidized triacylglycerols in canola oil and margarine by LC-MS, J. Agric. Food Chem. 67 (2019) 10174-10184. <https://doi.org/10.1021/acs.jafc.9b03601>.
